# Inhibiting Vanadium Dissolution of Potassium Vanadate for Stable Transparent Electrochromic Displays

**DOI:** 10.1002/smsc.202300046

**Published:** 2023-07-13

**Authors:** Bin Wang, Feifei Zhao, Wu Zhang, Changyu Li, Kun Hu, Brett N. Carnio, Linhua Liu, William W. Yu, Abdulhakem Y. Elezzabi, Haizeng Li

**Affiliations:** ^1^ Institute of Frontier and Interdisciplinary Science Shandong University Qingdao Shandong 266237 China; ^2^ School of Chemistry & Chemical Engineering Shandong University Jinan Shandong 250100 China; ^3^ Ultrafast Optics and Nanophotonics Laboratory Department of Electrical and Computer Engineering University of Alberta Edmonton Alberta T6G 2V4 Canada

**Keywords:** dead Zn^2+^ sites, hybrid electrolytes, potassium vanadium oxide, transparent electrochromic displays, vanadium dissolution

## Abstract

Vanadium oxides are highly valued as electrochromic materials because of their multicolor capabilities. However, their practical applications have been limited due to challenges such as the dissolution of vanadate into aqueous electrolytes, leading to poor long‐term stability. Herein, a solution is proposed to the vanadate dissolution issue by utilizing a hybrid electrolyte consisting of tetraethylene glycol dimethyl ether (TEGDME) and water. This electrolyte has the unique ability to form a robust cathode electrolyte interface layer on vanadium oxide electrodes. As a proof of concept, zinc‐anode‐based multicolor transparent electrochromic displays are prepared using layered potassium vanadate (K_2_V_6_O_16_·1.5H_2_O, KVO) with a TEGDME–water hybrid electrolyte. By soaking the KVO electrode in the hybrid electrolyte, it is demonstrated that KVO has remarkable stability against dissolution. Furthermore, it is shown that KVO has superior electrochromic performance compared to sodium vanadate (NaV_3_O_8_·1.5H_2_O, SVO), due to the wide KVO interlayer spacing. Given the enhanced performance of this hybrid electrolyte and KVO cathode material, a zinc‐anode‐based electrochromic display prototype is shown to exhibit compelling performance. As such, this work is expected to be a significant catalyst for accelerating the development of vanadate‐based electrochromic displays.

## Graphical abstract

Potassium vanadate (KVO) nanorods having wide interlayer spacing are prepared and employed for electrochromic applications. By utilizing an ether–water hybrid electrolyte in a Zn–KVO electrochromic platform, vanadium dissolution of KVO is inhibited. These findings represent a promising paradigm for stable, transparent electrochromic displays.

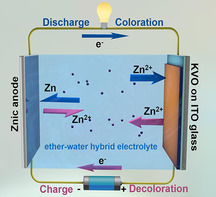

## 1 Introduction

Electrochromic technology exhibits many attractive characteristics (e.g., low power consumption, bistable characteristic, ease of fabrication, etc.) and has shown great potential for use in nonemissive displays,^[^ ^1^ ^]^ portable electronic devices,^[^ ^2^ ^]^ and smart windows.^[^ ^3^ ^]^ Recently, nonemissive electrochromic displays have attracted growing interest in both industry and academia, due to their insensitive viewing angle behavior, capability of retaining colored states without the need for external electrical power, and eye protection functionality.^[^ ^1^, ^4^, ^5^ ^]^ Nonetheless, the limited color range of a single electrochromic material has restricted the widespread acceptance of electrochromic displays, such that electrochromic displays exhibiting a richer color palette remain a great challenge within the electrochromic community.

With the integration of metamaterials into electrochromic devices being observed recently, there has been an acceleration in the development of electrochromic displays with a wide gamut of colors.^[^ ^6^, ^7^, ^8^ ^]^ However, using such materials has come at the cost of sacrificing device transparency, significantly restricting the application of these displays.^[^ ^9^, ^10^ ^]^ To address such issues, our previous works reported on zinc‐anode‐based electrochromic displays having 2D CIE color space tunability, which offers a promising strategy to realize transparent electrochromic displays with broadened color hues.^[^ ^5^, ^11^ ^]^ These electrochromic display platforms utilized the color overlay effect of a bipolar coloring electrochromic material, with SVO being employed, as vanadium oxides are considered the most promising inorganic material for multicolor electrochromic displays.^[^ ^12^, ^13^ ^]^ Although sodium ions positioned within the V_3_O_8_ layers broaden the interlayer spacing and serve as pillars to stabilize the tunnel structure during Zn^2+^ insertion/extraction,^[^ ^14^, ^15^ ^]^ the SVO electrochromic electrode nonetheless experiences Zn^2+^ traps (i.e., “dead Zn^2+^ sites”). The SVO electrode shows irreversible redox reactions, resulting in poor long‐term stability during cycling. An even more pressing challenge associated with vanadate‐based electrodes has been in the dissolution of SVO into an aqueous solution, which is a primary concern when using an aqueous electrolyte with the aforementioned zinc‐anode‐based electrochromic display platforms.^[^ ^16^, ^17^ ^]^

While the zinc‐anode‐based electrochromic display platform is still in its infancy, exploring multicolor electrochromic materials and developing electrochromic displays exhibiting long‐term stability warrant further investigation. The key interest is to improve the reversibility of Zn^2+^ insertion into vanadate and Zn^2+^ extraction from vanadate. One strategy to promote the Zn^2+^ reversibility is designing vanadate platforms having a broad interlayer spacing. It is desirable to apply KVO as the electrochromic material for constructing transparent electrochromic displays, given the larger ionic radius of K^+^ (≈1.38 Å) compared to other alternatives (e.g., Na^+^, ionic radius ≈1.02 Å). Here, the larger K^+^ radius is expected to expand the space between the V_3_O_8_ layers, thus suppressing the formation of Zn^2+^ traps (i.e., “dead Zn^2+^ sites”). To date, there have been no reports on the utilization of KVO for electrochromic devices.

It has been shown that the addition of ether could boost the formation of a robust and uniform cathode electrolyte interface (CEI) film on the vanadate cathodes, thereby inhibiting the dissolution of vanadium species.^[^ ^18^, ^19^ ^]^ Unfortunately, the employment of an ether–water hybrid electrolyte for vanadate‐based electrochromic devices is unexplored. To address the dissolution issues of vanadate electrodes in aqueous electrolytes, ether–water hybrid electrolytes could be incorporated into Zn–KVO transparent electrochromic displays.

Herein, we present a KVO cathode that effectively reduces the formation of “dead Zn^2+^ sites” during electrochromic processes. Furthermore, the use of a hybrid electrolyte consisting of tetramethylene glycol dimethyl ether (TEGDME) and water significantly inhibits the dissolution of the KVO electrochromic cathode. In comparison to an SVO cathode, the KVO cathode has a wider interlayer spacing and can extract almost all of the inserted Zn^2+^ during the charging process. In contrast, only 9.37% of inserted Zn^2+^ are extracted from a discharged (i.e., green‐colored) SVO cathode when switching its color from green to orange. The KVO electrode exhibits rapid switching times of 12.9/16.9 s for coloration and bleaching, respectively, whereas the SVO electrode requires 23.4/28.9 s for the same processes. Additionally, the KVO electrode demonstrates stability in a TEGDME–water hybrid electrolyte for 28 d, suggesting a potential approach for constructing long‐lasting vanadate electrochromic electrodes. As a proof of concept, the enhanced electrochromic performance is demonstrated in a zinc anode‐based transparent electrochromic display prototype, which shows compelling energy retrieval functionalities and fascinating multicolors arising from the color overlay effect.

## 2 Results

### 2.1 Characterization of KVO Nanorods

**Figure 1:**
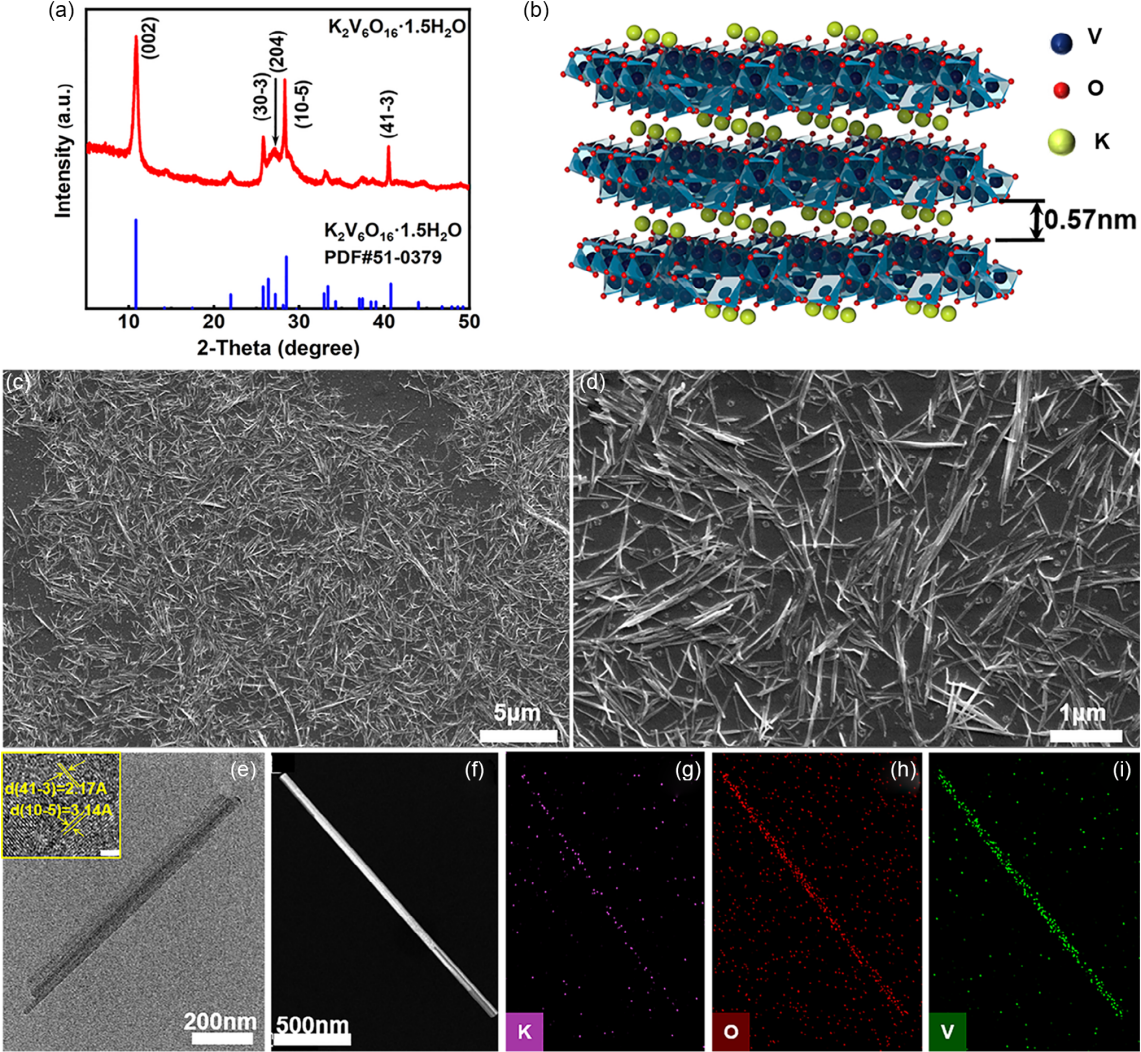
Characterization of KVO nanorods. a) XRD pattern of the as‐prepared KVO nanorods. b) Illustration of the KVO crystal structure. c) Low‐magnification and d) high‐magnification SEM images of the KVO nanorods. e) TEM image of a single KVO nanorod (inset: high‐resolution TEM image of a KVO nanorod depicting the lattice planes (scale bar: 2 nm)). f–i) Dark‐field TEM image of a single KVO nanorod and the corresponding elemental mapping images of K, O, and V.

The KVO nanorods were synthesized at room temperature according to a modified liquid–solid stirring method (see details in the “Experimental Section”).^[^ ^11^ ^]^ The KVO powder, obtained by centrifugation and dried at room temperature, is analyzed for its phase composition using X‐ray diffraction (XRD). As depicted in the XRD pattern **(** **Figure** **1**a **)**, the KVO powder diffraction peaks are perfectly indexed to the monoclinic K_2_V_6_O_16_·1.5H_2_O phase (Joint Committee on Powder Diffraction Standards No. 51‐0379). These monoclinic phase KVO nanorods have a layered structure, similar to the SVO nanorods reported in our previous work.^[^ ^11^ ^]^ As illustrated in Figure 1b, the crystal structure of KVO contains hydrated potassium ions embedded within the V_3_O_8_ layers, which stabilize the structure during electrochromic processes. Due to the larger ionic radius of K^+^ in comparison to Na^+^, the V_3_O_8_ interlayer spacing of KVO nanorods (0.537 nm) is 5.29% broader than that of SVO nanorods (0.51 nm, Figure S1, Supporting Information). Such enlarged interlayer spacing improves Zn^2+^ insertion/extraction and diminishes lattice deformation during cycling, resulting in rapid switching and good cycle stability. To reveal the rod‐like morphology of KVO, scanning electron microscope (SEM) imaging is employed. Figure 1c,d depicts the morphology of KVO at distinct regions and magnifications, respectively, revealing that the KVO nanorods are 0.56–1.3 μm in length and 30–50 nm in diameter (Figure 1c, S2, and Table S1, Supporting Information). To investigate the aspect ratio and crystallization of the KVO nanorods, transmission electron microscopy (TEM) is employed, showing that the KVO nanorod exhibits an average aspect ratio of 23.25 (Figure 1e). The high‐resolution TEM image (inset in Figure 1e) shows crystalline lattice spacings of 0.217 and 0.314 nm for the (41‐3) and (10‐5) crystal planes, respectively, further confirming the preparation of monoclinic K_2_V_6_O_16_·1.5H_2_O. Energy‐dispersive X‐ray (EDX) spectroscopy mapping images of a single KVO nanorod are presented in Figure 1f–i, revealing the existence and uniform distribution of V, O, and K elements. The relatively low potassium fraction observed in EDX mapping is consistent with the proportion of potassium atoms in monoclinic K_2_V_6_O_16_·1.5H_2_O (Table S2, Supporting Information).

### 2.2 Inhibition of KVO Dissolution by Organic–Water Hybrid Electrolytes

**Figure 2:**
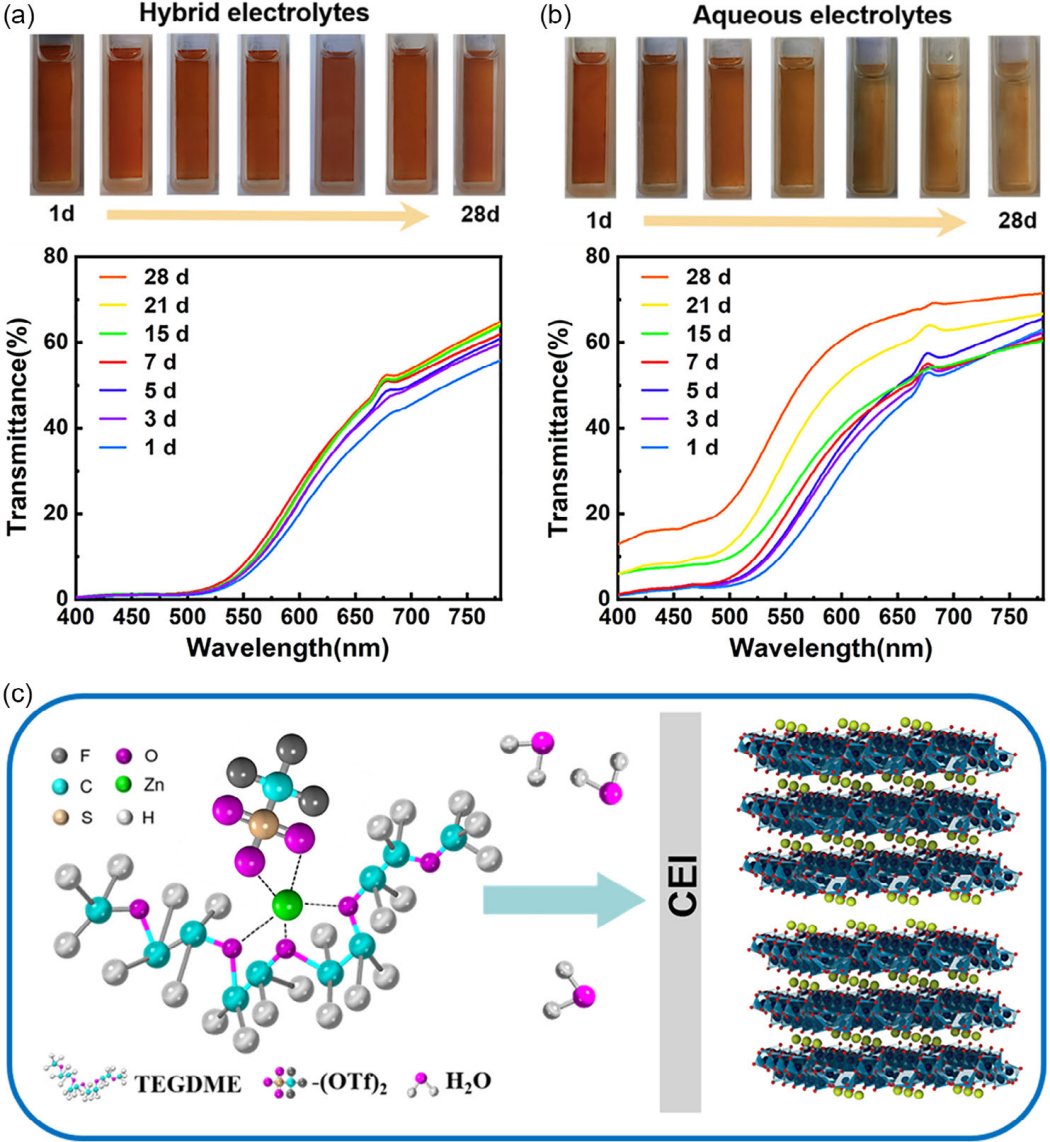
Organic–water hybrid electrolytes inhibiting the dissolution of KVO electrodes. a,b) Optical transmittance spectra and digital photographs of a KVO electrode immersed in the hybrid electrolyte (a) and the aqueous electrolyte (b) at different aging times. c) A schematic diagram of the corresponding Zn^2+^ solvation structure, as well as the CEI membrane. This schematic diagram is drawn based on information from ref. [18].

After successfully preparing KVO with a broad interlayer spacing, the KVO dissolubility is investigated in different electrolytes. The long pair of oxygen atoms within the structures of ether‐based solvents can coordinate with Zn^2+^ and provide H‐bond acceptors to bond with H_2_O, thus limiting the reductive activity of water.^[^ ^19^ ^]^ TEGDME, which has five ether groups (O_ether_) in its molecular chain, is expected to effectively coordinate with Zn^2+^ and suppress water‐induced parasitic reactions.^[^ ^19^ ^]^ To test this hypothesis, we mix TEGDME with the most commonly used aqueous electrolyte (i.e., 0.5 m Zn(OTf)_2_ aqueous electrolyte). A KVO electrode is immersed in an aqueous electrolyte (i.e., 0.5 m Zn(OTf)_2_ in water), and an identical KVO electrode is immersed in a hybrid electrolyte (i.e., 0.5 m Zn(OTf)_2_ in a TEGDME–water mixture (1:4 volume ratio)). As shown in **Figure** **2**a, the KVO electrode in the hybrid electrolyte exhibits no significant decay in optical transmittance or any other apparent changes after 28 d. In contrast, the KVO electrode gradually dissolved into the pure aqueous electrolyte, significantly increasing the electrode's transmittance (Figure 2b). To quantitatively compare the different dissolution performances of the KVO electrodes in hybrid and pure aqueous electrolytes, two identical KVO electrodes are soaked in these electrolytes for seven days. The electrolytes are then collected and analyzed using inductively coupled plasma atomic emission spectrometry (ICP‐AES). As indicated by the ICP‐AES spectra (Figure S3, Supporting Information), the concentration of dissolved vanadium in the hybrid electrolyte (≈0.93 ppm) is much lower than that in the pure aqueous electrolyte (≈7.26 ppm), clearly revealing that the hybrid electrolyte effectively inhibits the dissolution of the KVO film. Previous literature has attributed the inhibition of vanadate dissolution to:^[^ ^18^ ^]^ 1) the TEGDME component coordinating with water to diminish the water's activity and 2) the TEGDME preferentially amassing at the electrode–electrolyte interface, leading to a robust CEI layer that suppresses dissolution of vanadium species. Therefore, the inhibited KVO dissolution observed in this work is conceivably due to the reduction of water's activity and the formation of a robust CEI layer on the KVO electrode (Figure 2c). TEM imaging is conducted to further investigate the CEI layer. As shown in Figure S4, Supporting Information, the CEI layer remains after 100 coloring–bleaching cycles, suggesting that the as‐formed CEI layer is robust as it endures the coloring–bleaching cycles. Despite these useful observations, a more detailed investigation of the origin of KVO dissolution inhibition remains a topic to be addressed in future works.

### 2.3 Electrochromic Performance of the KVO Electrodes

**Figure 3:**
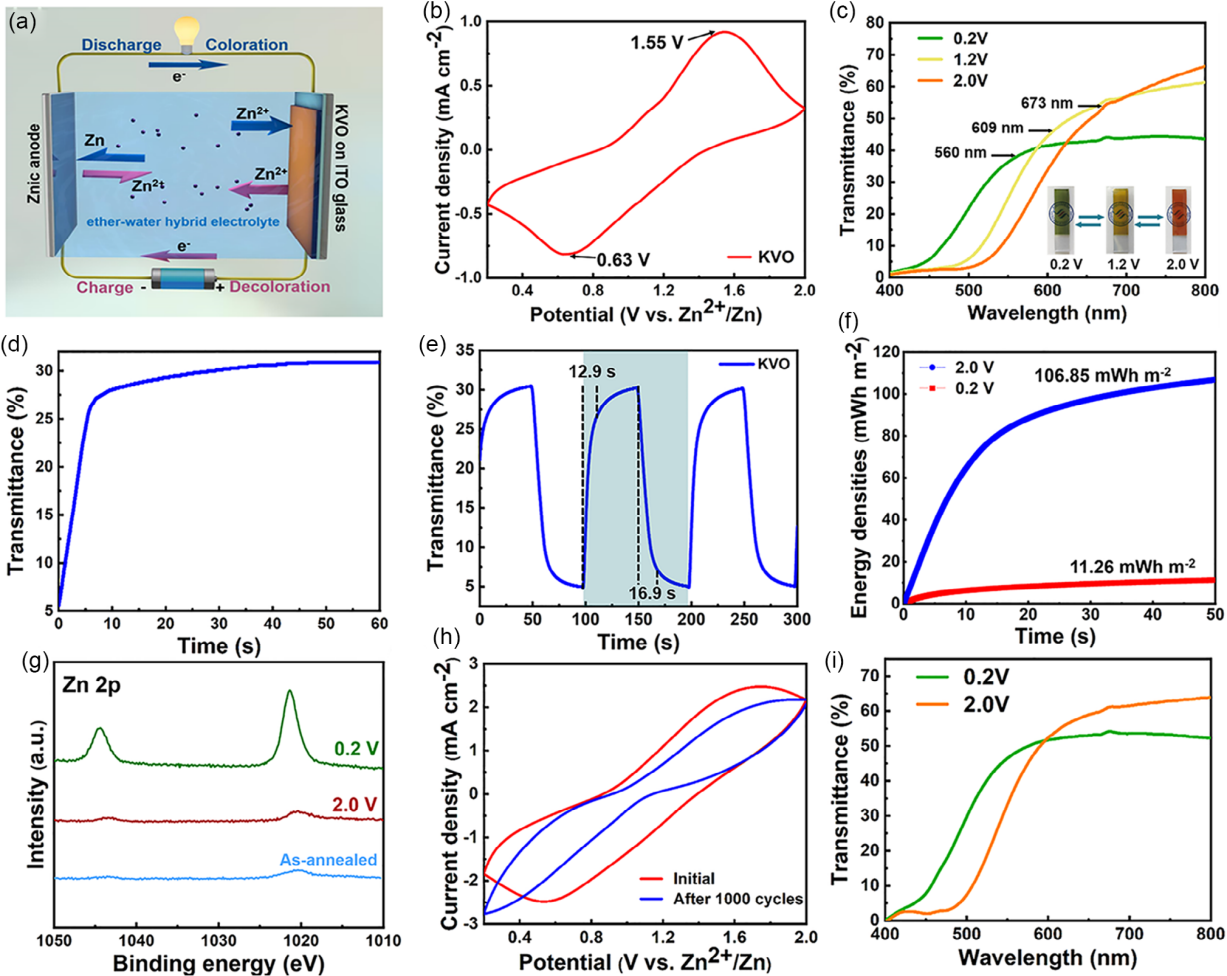
Electrochromic performance of the KVO electrodes. a) Schematic diagram of the Zn–KVO electrochromic device. b) Cyclic voltammograms of the KVO electrodes at a scan rate of 50 mV s^−1^ over a voltage range of 0.2–2.0 V. c) Optical transmittance spectra of the KVO electrodes at different applied voltages. Inset: corresponding digital photos of the KVO electrodes. d) In situ self‐coloring process (spontaneous color switching from orange to green) of the KVO electrode. e) Dynamic test of the KVO electrode at 521 nm in the 0.2–2.0 V window. f) Round‐trip energy density comparison of the KVO electrode during the selected cycle (dark‐green region in (e)) of the dynamic test. g) Ex situ XPS Zn 2p survey spectra of the KVO electrode during pristine, charged, and discharged states. h) CV measurement of the KVO electrodes over 1000 cycles between 0.2 and 2.0 V at 150 mV s^−1^. i) Visible–near‐infrared transmittance spectra of the KVO electrode after 1000 CV cycles.

With the KVO electrode confirmed to have a wide interlayer spacing and the hybrid electrolyte shown to prevent KVO dissolution, we next investigate the enhanced electrochromic performance of KVO electrodes. The electrochromic performance of KVO electrodes is compared to that of SVO electrodes. Electrochemical measurements are conducted using a two‐electrode configuration, incorporating zinc metal as the anode and vanadate electrodes as the cathode. The electrolyte used in such a platform is prepared by dissolving 0.5 m Zn(OTf)_2_ into a mixture of TEGDME and distilled water (1:4 in volume). The Zn–KVO two‐electrode configuration could provide several key advantages, including excelling energy retrieval functionalities and simplified counter electrode design.^[^ ^20^ ^]^ The working mechanism of this configuration is shown in **Figure** **3**a, illustrating the redox reactions during the spontaneous color switching (discharge) process and color recovery (charging) process.

A pair of KVO reduction and oxidation peaks (occurring at ≈0.63/1.55 V, respectively) are observed in the cyclic voltammetry (CV) curve (Figure 3b). The reduction peak potential (0.63 V) of the KVO electrode is higher than that of the SVO electrode (0.54 V, Figure S5a, Supporting Information), while the oxidation peak potential (1.55 V) is lower than that of the SVO electrode (1.773 V, Figure S5a, Supporting Information). As such, in comparison to the SVO electrode, the KVO electrode is more amenable to being reduced and oxidized when subjected to external voltages. Figure 3c shows the change in the optical transmittance spectra of the KVO electrode under different applied voltages. The inset in Figure 3c reveals that the KVO electrode exhibits noticeable color changes (orange ⇄ yellow ⇄ green) when applying different voltages. The color switch from orange to green results in a 113 nm blueshift of the transmission peak (673–560 nm), which is wider than the shift seen from our previously reported SVO electrode.^[^ ^11^ ^]^ The wider spectral regulation range of the KVO electrode marks its great promise in display applications, as it exhibits more color hues. The KVO electrode also demonstrates a high transparency (≈50%) and an optical contrast of 25.3% at 521 nm, which is comparable to previous reports.^[^ ^12^, ^21^, ^22^ ^]^

As zinc‐anode‐based electrochromic devices possess the additional advantage of self‐coloration,^[^ ^23^ ^]^ we investigate the spontaneous color‐switching performance of the Zn–KVO electrochromic platform. The spontaneous color switching time, defined as the time required to achieve 90% of the maximum optical contrast,^[^ ^24^ ^]^ needed for the KVO electrode to adjust from orange to green is measured to be 8.8 s (Figure 3d). As shown in Figure 3e, the KVO electrode switching time required when changing from orange to green is 12.9 s, while the recovery time when changing from green to orange is 16.9 s. These times are much more rapid than those of the SVO electrode (23.4/28.9 s, Figure S5b, Supporting Information), attributed to the broad interlayer spacing of the KVO nanorods, the large KVO electrode reduction peak potential, and the small KVO electrode oxidation peak potential. Important to note is that even faster switching times are envisioned to be realized by using hybrid Zn^2+^/M^+^ (M^+^ denotes Li^+^, Na^+^, Al^3+^, etc.) electrolytes.^[^ ^3^, ^23^, ^25^ ^]^ Additionally, the electrochromic performance of KVO electrodes, using hybrid ether–water gel electrolytes, is superior to that of a pure water gel electrolyte (Figure S6, Supporting Information), indicating that hybrid ether–water electrolytes are of significant promise for nonleakage practical applications.

A primary advantage of the newly established Zn anode‐based electrochromic devices is that the Zn–KVO electrochromic platform eliminates the need for external energy during the coloration process.^[^ ^20^ ^]^ As the Zn–KVO electrochromic platform is able to effectively retrieve the consumed energy during the spontaneous coloring process (similar to the discharge process of secondary batteries), we evaluate the net round‐trip energy consumption. The energy consumption of the KVO electrode during the bleaching process is calculated to be 106.35 mWh m^−2^, while 11.26 mWh m^−2^ of the consumed energy is retrieved during spontaneous color switching from orange to green, as presented in Figure 3f. This corresponds to a net round‐trip energy consumption of 95.09 mWh m^−2^, which is superior to that of TiO_2_‐based electrochromic devices^[^ ^22^ ^]^ and comparable to Zn–SVO platforms incorporating an aqueous electrolyte.^[^ ^11^ ^]^ Aside from this enhanced energy retrieval performance, the coloration efficiency (CE) of the KVO electrode is improved in comparison to the SVO electrode. As depicted in Figure S7, Supporting Information, the CE of the KVO electrode is calculated as 62.08 cm^2^ C^−1^, higher than that of the SVO electrode (44.58 cm^2^ C^−1^).

It is well known that “dead cation sites” formed during cycling result in the degradation of electrochromic performance and poor reversibility,^[^ ^26^ ^]^ making it crucial to eliminate the formation of “dead cation sites.” To test our hypothesis that the broad KVO interlayer spacing could significantly eliminate the “dead Zn^2+^ sites” formed during cycling, we investigate the ex situ X‐ray photoelectron spectroscopy (XPS) spectra of the KVO electrode under different color states **(**Figure S8, Supporting Information). As shown in Figure 3g, the as‐annealed orange KVO electrode exhibits no significant amount of the Zn element. In contrast, the green‐colored (i.e., 0.2 V‐discharged) KVO electrode shows obvious Zn element peaks, suggesting that color switching from orange to green is due to the insertion of Zn^2+^ into the KVO lattice. Surprisingly, the Zn 2p XPS spectrum of the 2.0 V‐charged KVO electrode reveals that almost all inserted Zn^2+^ cations are extracted when switching the KVO electrode from green to orange. In comparison, the SVO electrode shows inferior reversibility, as only ≈9.37% (calculation process outlined in Note S1, Supporting Information) of the inserted Zn^2+^ are extracted by applying a 2.0 V external voltage (Figure S9, Supporting Information). These results confirm that KVO nanorods, having a wide interlayer spacing, are effective in eliminating the electrostatic interactions between Zn^2+^ and V_3_O_8_ lattice, thus suppressing the formation of “dead Zn^2+^ sites.”

To shed light on the cycling performance of the KVO electrode in the hybrid electrolyte, we record the KVO electrode capacity evolution and optical contrast retention after 1000 CV cycles. As shown in Figure 3h, the KVO electrode retains 55.86% of its initial capacity in the hybrid electrolyte after 1000 cycles, but shows no apparent electrochemical activity in a pure aqueous electrolyte (with and without the addition of PVA) after 1000 cycles (Figure S10a–c, Supporting Information). This indicates that the TEGDME–water hybrid electrolyte is a promising paradigm for long‐term vanadate‐based electrochromic devices, due to their ability to inhibit vanadium dissolution. Additionally, the KVO electrode exhibits better cycling stability in comparison to the SVO electrode in the hybrid electrolyte (15.38% capacity retention after 1000 cycles, Figure S10d, Supporting Information), further confirming that KVO having wider interlayer spacing eliminates “dead Zn^2+^ sites” and improves reversibility. The KVO electrode maintains 89% of its initial optical contrast after 1000 cycles (Figure 3i), which is significantly higher than that of the SVO electrode (2.06%, Figure S10e, Supporting Information). Likewise, the cycling stability of the Zn–KVO platform incorporating the hybrid electrolyte is superior to that of previously reported vanadate‐based electrochromic devices,^[^ ^27^, ^28^, ^29^ ^]^ further affirming that designing high‐performance electrolyte systems should be a primary task undertaken by the electrochromic community.

### 2.4 Demonstration of Zn–KVO Electrochromic Displays

**Figure 4:**
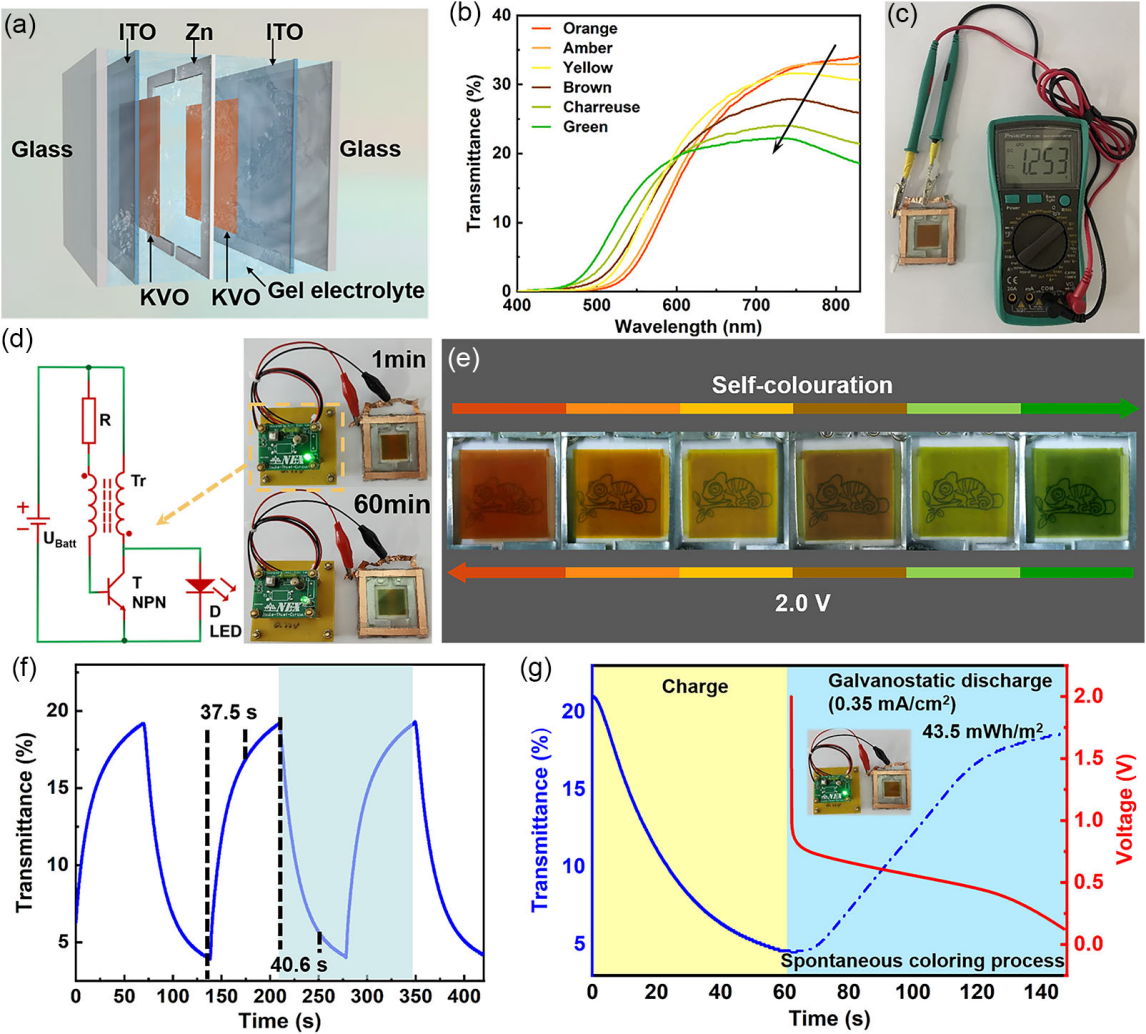
Electrochromic performance of the Zn–KVO electrochromic display prototype. a) Schematic diagram of the KVO–Zn–KVO electrochromic display configuration. b) Optical transmittance spectra of the KVO–Zn–KVO electrochromic display at different applied voltages. c) Digital photograph of the orange‐colored KVO–Zn–KVO electrochromic display, showing an OCP of 1.25 V. d) Digital photograph of a 0.2 V regulated LED powered by the KVO–Zn–KVO electrochromic display at 1 and 60 min, connected via the “Joule thief circuit”. e) Digital photographs of the KVO–Zn–KVO transparent electrochromic display exhibiting six colors via the color overlayer effect, where the cartoon beneath the display is seen by the naked eye. f) Dynamic test of the KVO–Zn–KVO electrochromic display at 521 nm in the 0.2–2.0 V electrochemical window. g) Transmittance at the wavelength of 521 nm during the chronoamperometric charging process at 2.0 V (solid blue line) and the galvanostatic discharge process at a current density of 0.35 mA cm^−2^ (dotted blue line). The red line is the corresponding galvanostatic discharge curve, and the inset photograph shows an LED (0.2 V regulated) lit up by the KVO–Zn–KVO display.

To demonstrate the applicability of the Zn–KVO electrochromic platform, a 5 × 5 cm transparent multicolor electrochromic display is assembled, as depicted in **Figure** **4**a. As KVO electrodes having a high electrochromic performance are realized when using a hybrid ether–water gel electrolyte (Figure S6, Supporting Information), the prototype electrochromic display is assembled using this electrolyte. Such a KVO–Zn–KVO platform could enable a richer color palette via the color overlay effect of two segments of the KVO electrodes.^[^ ^11^ ^]^ As a single KVO electrode has three intrinsic colors (i.e., orange, yellow, and green; Figure 3c), the KVO–Zn–KVO display utilizing the color overlay effect could easily realize six colors. Figure 4b shows the change in the optical transmittance of the KVO–Zn–KVO display under six color states (i.e., orange, amber, yellow, brown, chartreuse, and green). Color switching from orange to green results in a 174 nm blueshift of the transmission peak (776–602 nm), while the display maintains a semitransparency of >20%. The orange‐colored KVO–Zn–KVO display possesses a built‐in voltage (i.e., open‐circuit potential, OCP) of 1.25 V (Figure 4c), due to the redox potential difference between the zinc anode and the KVO cathode.^[^ ^30^ ^]^ This OCP enables the KVO–Zn–KVO display to spontaneously switch color from orange to green, accomplished by connecting the display to a 0.2 V regulated LED through the “Joule thief circuit” for 60 min (Figure 4d), with this switching process being similar to that observed with secondary batteries. The vivid colors of a single KVO–Zn–KVO electrochromic display are shown in Figure 4e. Due to the display's excellent semitransparency, the cartoon beneath the display is clearly visible to the naked eye. The dynamic transmittance characteristics of the KVO–Zn–KVO electrochromic display are evaluated in the 0.2–2.0 V window (shown in Figure 4f), where the switching times are calculated as 37.5 s for coloration and 40.6 s for bleaching. The inferior switching times of the display, in comparison with the single electrode using liquid electrolytes (Figure 3e), are attributed to the employment of the gel electrolyte, resulting in a decay of the cations transportation speed. To investigate the energy efficiency of the KVO–Zn–KVO electrochromic display during a color‐switching round trip, we determine the input energy during the chronoamperometric charging process (when applying a voltage of 2.0 V for 60 s) and the retrieved energy during the galvanostatic discharging process at a current density of 0.35 mA cm^−2^. The high current density of 0.35 mA cm^−2^ is chosen because it allows the orange‐colored KVO–Zn–KVO electrochromic display to spontaneously switch to green more rapidly. As shown in Figure S11, Supporting Information and Figure 4g, the input energy during the chronoamperometric charging process is calculated as 111.6 mWh m^−2^, while the retrieved energy during the galvanostatic discharging process is 43.5 mWh m^−2^. As the round‐trip energy efficiency (38.98%) presented in this work is superior to that of the previous report,^[^ ^11^ ^]^ it seems clear that the coming age of KVO–Zn–KVO electrochromic displays using a TEGDME–water hybrid electrolyte is appearing on the near horizon.

## 3 Conclusion

This is the first demonstration showing KVO nanorods prepared using a simple solution method, with the nanorods employed as an electrode for electrochromic devices. The KVO electrode exhibited outstanding electrochromic performance and significantly eliminated the formation of “dead Zn^2+^ sites,” attributed to its wide interlayer spacing. Utilizing a TEGDME–water hybrid electrolyte significantly inhibited the dissolution of vanadium species, likely due to reduced water activity and the formation of a CEI layer on the KVO electrode. By employing the well‐designed KVO electrode and TEGDME–water hybrid electrolyte, a KVO–Zn–KVO electrochromic display prototype was shown to be capable of switching between six colors (i.e., orange, amber, yellow, brown, chartreuse, and green) along with compelling energy retrieval functionality. The enhanced electrochromic performance detailed in this article is expected to accelerate the development of transparent electrochromic displays.

## 4 Experimental Section

4.1

4.1.1

##### Materials

All the chemicals were of analytical grade and were used without further purification. Zinc triflate (Zn(CF_3_SO_3_)_2_, Zn(OTf)_2_, 98%), zinc foil (Zn, 99.9%), vanadium oxide (V_2_O_5_, 99%), potassium chloride (anhydrous, 99%), and poly(vinyl alcohol) (PVA, *M*_w_ ≈195 000) were purchased from Macklin Biochemical Technology Co. Ltd. Hydroxyethyl cellulose (HEC) was purchased from Usolf Chemical Co. Ltd. and ITO glass was purchased from Zhuhai Kaivo Glass Co. Ltd.

##### Synthesis of KVO Nanorods

First, 100 g of commercial V_2_O_5_ powder was added into 1.5 L of a KCl aqueous solution (2 m) at room temperature and stirred for 5 days to form a solution with a jacinth suspension. Second, the sample was washed with deionized water six times. Third, the product was diluted with distilled water to form a precursor solution (8 mg mL^−1^). Finally, the precursor solution was sonicated in an ultrasonic bath until a homogeneous KVO colloid was formed.

##### Fabrication of the Electrodes via a Bar‐Coating Method

A KVO paste of proper viscosity needed to be prepared before bar‐coating. Therefore, 1.4 g of HEC was added to 60 mL of the KVO colloid (8 mg mL^−1^) at 60 °C under stirring for 24 h. The ITO glass substrates were pretreated by acetone, ethanol, and deionized water sequentially and dried in ambient air. Next, the KVO/HEC paste was bar‐coated onto an ITO glass substrate to form a uniform film. After heat treatment of the KVO/HEC coated ITO glass, the KVO electrode having a thickness of ≈984 nm was prepared **(**Figure S12, Supporting Information).

##### Assembly of Electrochromic Displays

The Zn(OTf)_2_ solution was prepared by dissolving 0.5 m Zn(OTf)_2_ in a mixture of TEGDME–water (1:4 volume ratio). After that, 6 g of PVA was added to 60 mL of 0.5 m Zn(OTf)_2_ solution to form a gel electrolyte. The KVO–Zn–KVO electrochromic displays were constructed by sandwiching a thin Zn disconnected frame between two pieces of KVO electrodes. The PVA–Zn(OTf)_2_ gel was used as the electrolyte.

##### Characterization

The crystal structures and morphology of the samples were examined by XRD (Rigaku D/Max 2500PC diffractometer with a graphite monochromator and Cu Kα radiation (*λ* = 0.15418 nm)), XPS (PHI 5000 VersaProbe III), Field emission SEM (Quanta 250 FEG), TEM (FEI Tecnai G2 F20), UV–vis–near‐IR spectrophotometry (UH5700), and high‐resolution transmission electron microscopy (HRTEM) (FEI Talos F200X G2). The concentration of vanadium in the electrolytes was examined by the inductively coupled plasma atomic emission spectrometer (iCAP‐7400). All electrochemical measurements were carried out using an electrochemical workstation (CHI‐760E; CH Instruments, Shanghai, China) in a two‐electrode configuration, using the KVO electrode as the working electrode and Zn foil as both the counter electrode and reference electrode. In situ optical transmittance, as a function of the applied potential, was obtained in a quartz cuvette recorded by the UV–vis–near‐IR spectrophotometer. ITO glass in the electrolyte was used as the baseline for measuring the transmittance curve of the electrodes, which means that all the optical transmittance spectra were measured without subtracting the transmission loss due to the substrate.

## Conflict of Interest

The authors declare no conflict of interest.

## Supporting information

Supplementary Material

## Data Availability

The data that support the findings of this study are available from the corresponding author upon reasonable request.
