# Supplementary material for: Inhibiting Vanadium Dissolution of Potassium Vanadate for Stable Transparent Electrochromic Displays
Source: Small Sci. 2023 Jul 13;3(9):2300046. doi: 10.1002/smsc.202300046 (PMC11935851; doi:10.1002/smsc.202300046)
Supplement: Supplementary file 1 — Supplementary Material [file SMSC-3-2300046-s001.pdf]

Supporting Information

Inhibiting Vanadium Dissolution of Potassium Vanadate for Stable Transparent Electrochromic Displays

Bin Wang, Feifei Zhao, Wu Zhang, Changyu Li, Kun Hu, Brett N. Carnio, Linhua Liu, William W. Yu, Abdulhakem Y. Elezzabi, and Haizeng Li**

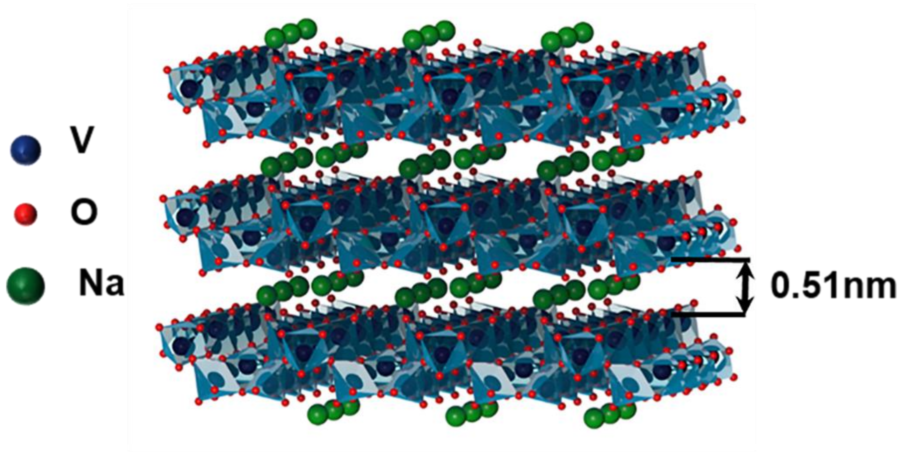

Figure S1. Schematic diagram of the SVO.

Figure S1 shows that SVO is a layered structure having Na^+ intercalated between the V_3O_8 interlayers, exhibiting a layer spacing of only 0.51 nm.

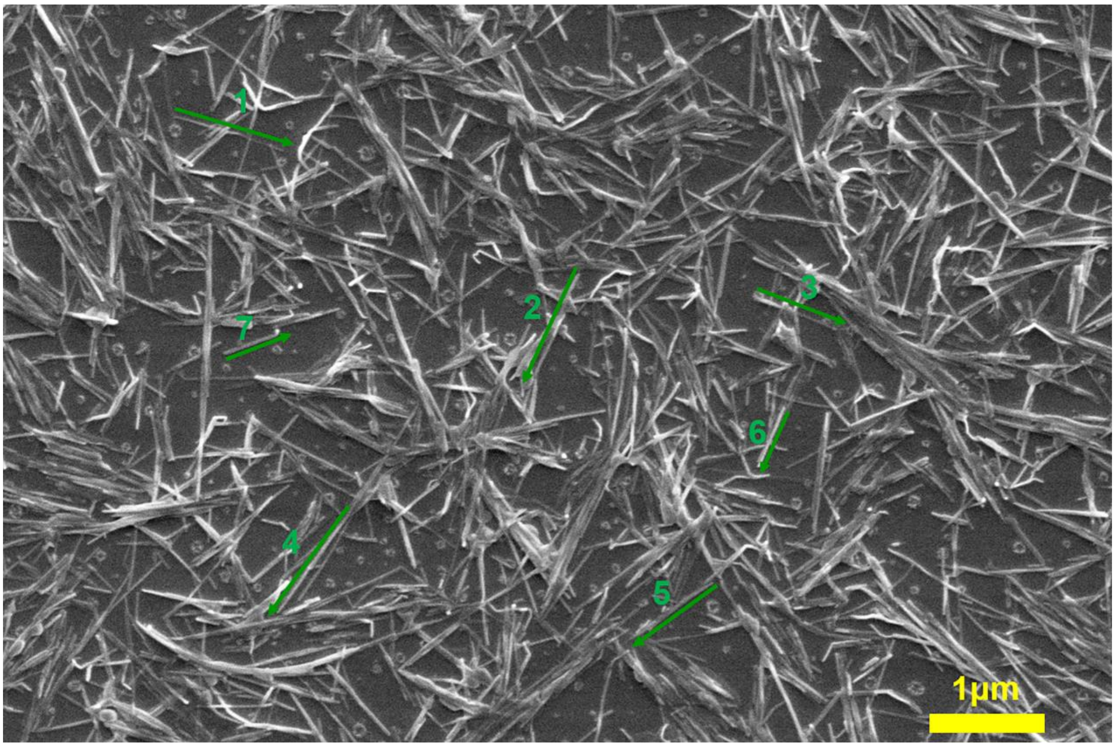

Figure S2. SEM image of the KVO nanorods.

Table S1. Diameter and length of the KVO nanorods.

Sample	Diameter(nm)	Length(μm)
1	30	1.3
2	40	1
3	37	0.86
4	30	1.1
5	50	1
6	30	0.63
7	40	0.56
**Range**	**30-50**	**0.56-1.3**

Figure S2 shows an SEM image of the KVO nanorods. Seven representative KVO nanorods are shown in Table S1, with specific diameters and lengths ranging from 30-50 nm and 0.56-1.3 μm, respectively.

Table S2. Atomic fraction of different elements in KVO nanorods.

Element	Atomic Fraction (%)
O	76.58
K	3.73
V	19.68

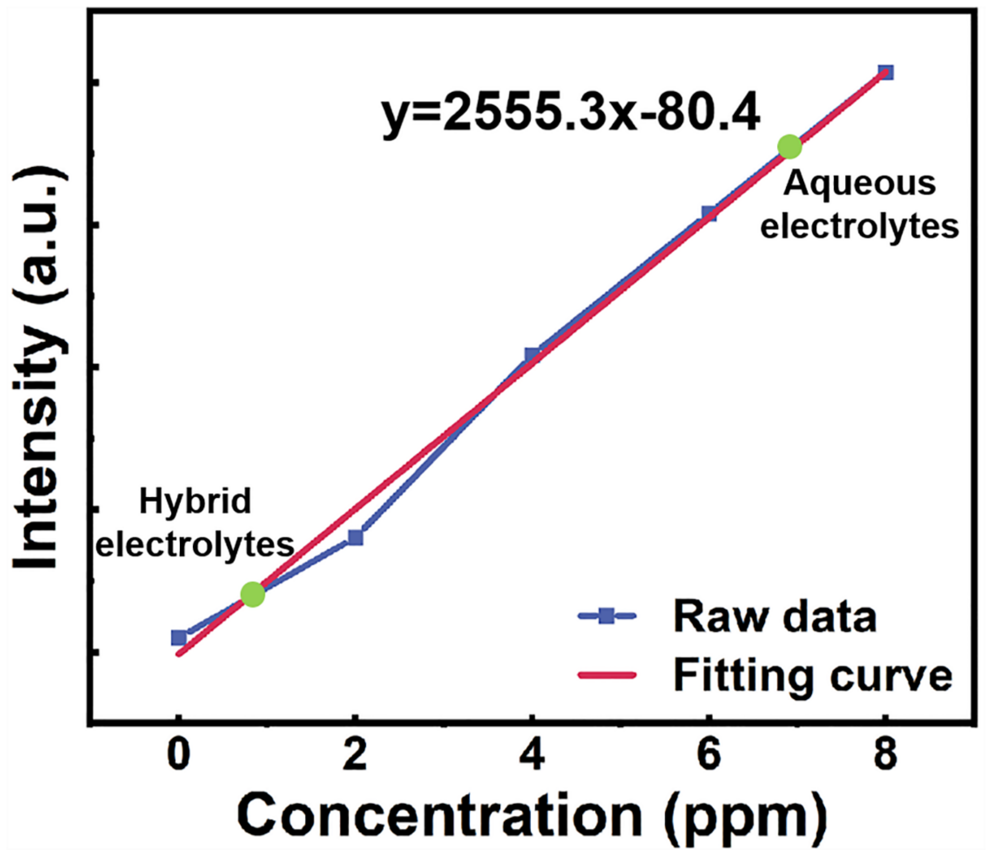

Figure S3. The spectrum of standard sample data and fitted equation.

According to the fitted standard equation (y=2555.3x-80.4) displayed in Figure S3, the vanadium concentrations in a pure aqueous electrolyte and a hybrid electrolyte are 7.26 ppm and 0.93 ppm, respectively.

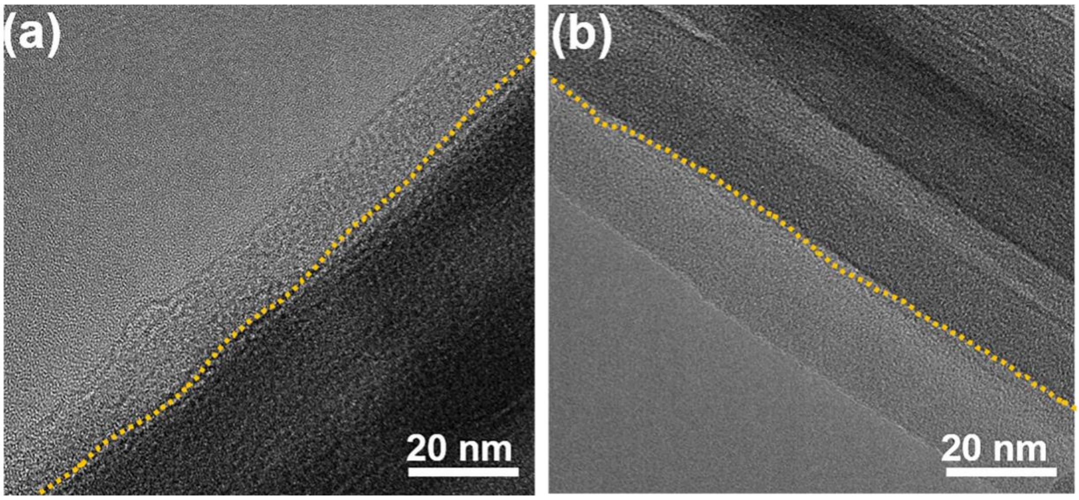

Figure S4. TEM images of the KVO electrode at the **(a)** 2nd and **(b)** 100th colored state in an ether-water hybrid electrolyte.

As shown in Figure S4a, the CEI layer is clearly presented at the 2nd colored state, which confirms the formation of this layer. The CEI layer is still observed after 100 coloring-bleaching cycles (Figure S4b), which reveals the excellent robustness of this layer.

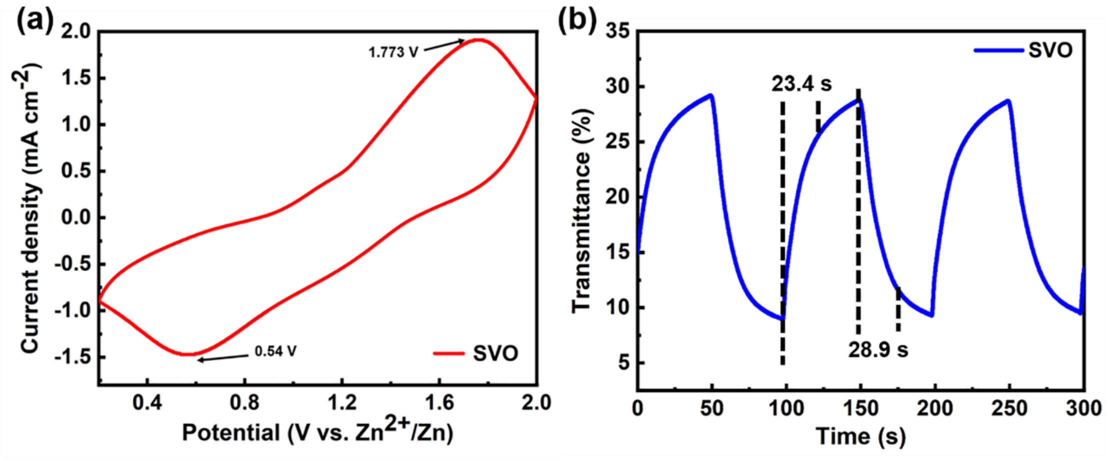

Figure S5. (a) Cyclic voltammogram (CV) measurement of the SVO electrodes at a scan rate of 50 mV/s. **(b)** The real-time transmittance spectra of the SVO electrode at 521 nm.

The CV curve of SVO electrodes is shown in Figure S5a. The pair of redox peaks for the SVO electrode is 1.78 and 0.54 V. The SVO switching times are shown in Figure S5b, exhibiting a coloration time (t_c) of 23.4 s and a bleaching time (t_b) of 28.9 s. The maximum optical contrast at 521 nm is determined as 19.4%, suggesting that the electrochemical performance of SVO is inferior to that of KVO (Figure 3e). This may be attributed to the larger radius of potassium ions compared to sodium ions, which broadens the KVO interlayer spacing.[1]

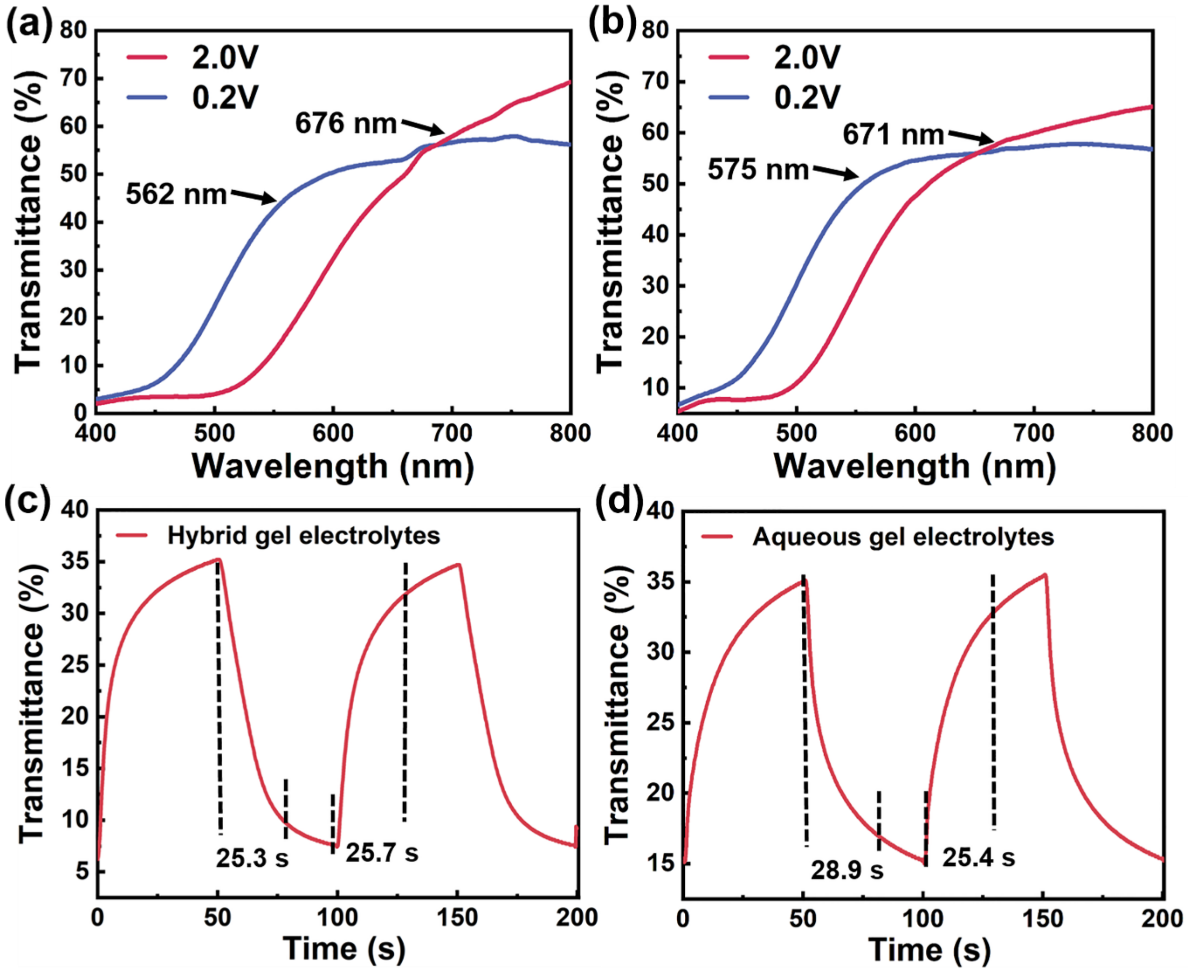

Figure S6. Optical transmittance spectra of the KVO electrode in a gel electrolyte system using **(a)** an ether-water mixture as the solvent and **(b)** pure water as the solvent, obtained at different applied voltages. The real-time transmittance spectra of the KVO electrode at 521 nm in a gel electrolyte system using **(c)** an ether-water mixture as the solvent and **(d)** pure water as the solvent.

As shown in Figure S6a, the KVO electrode could switch from orange to green with a 114 nm blueshift when being tested in a gel electrolyte using an ether-water mixture as the solvent. In contrast, the KVO electrode can only switch from orange to green with a 96 nm blueshift when being tested in a gel electrolyte using pure water as the solvent (Figure S6b). Although the switching times of the KVO electrodes in these two gel electrolytes (Figure S6c, d) are nearly identical, the KVO electrode in the hybrid gel electrolyte shows higher optical contrast (~27.6%) in comparison to the KVO electrode in the pure water gel electrolyte (~19.8%). This indicates that the hybrid ether-water gel electrolyte endows rapid switching speed. The aforementioned results affirm that utilizing an ether-water

mixture as the electrolyte solvent is a good candidate for building high-performance Zn-KVO electrochromic displays.

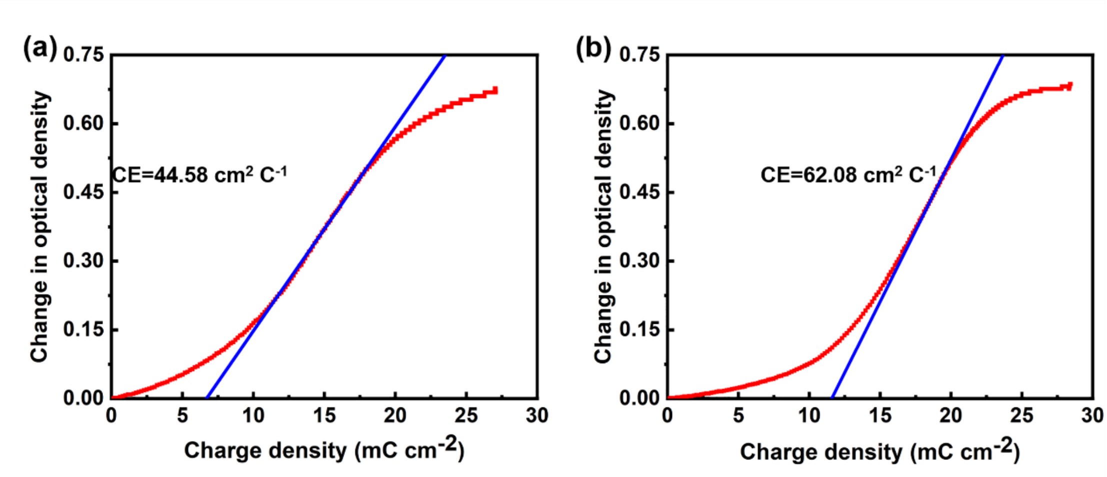

Figure S7. Coloration efficiency (CE) of the **(a)** SVO electrode and the **(b)** KVO electrode.

The coloration efficiency (CE) is closely related to ΔT and current density, with CE defined as the change in optical density (ΔOD) relative to the unit charge density (ΔQ) as observed during the coloring process.[2] As displayed in Figure S7, the CE of the SVO film and KVO film are 44.58 cm^2 C^{-1} and 62.08 cm^2 C^{-1}, respectively.

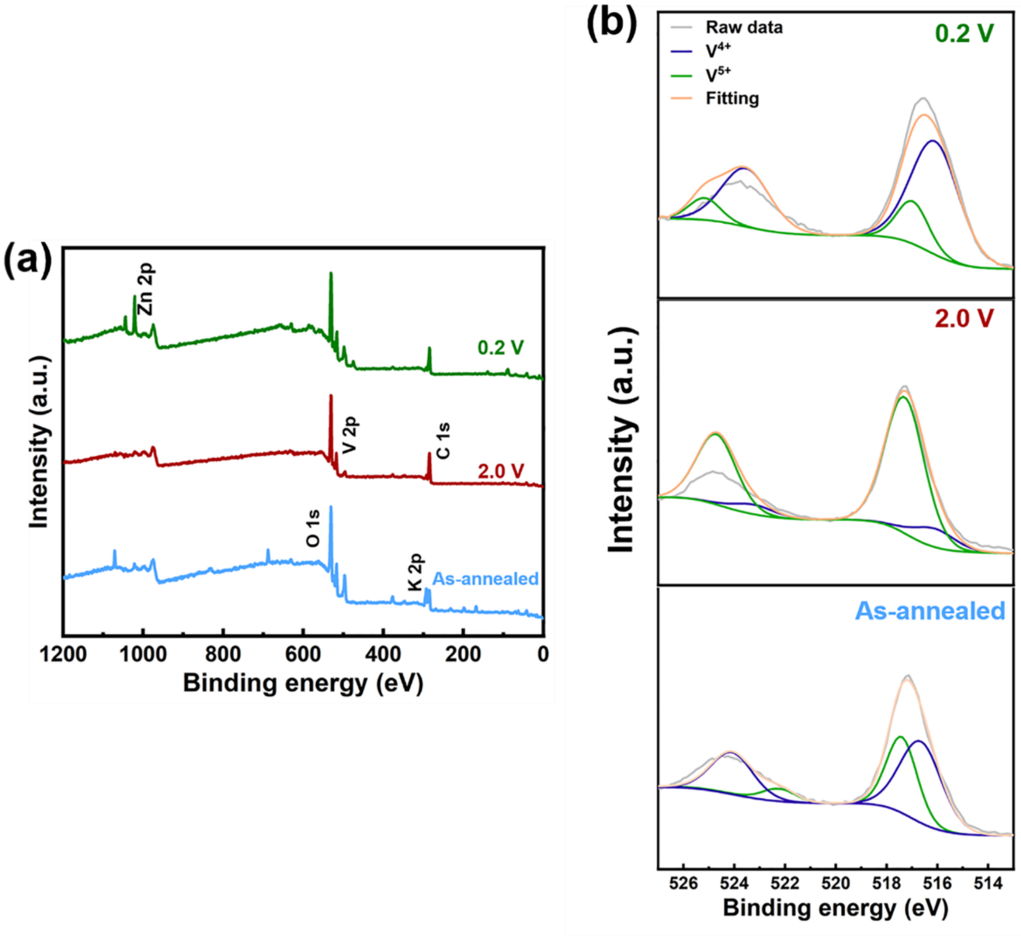

Figure S8. The ex-situ XPS survey spectra of the KVO electrode after being discharged/charged: **(a)** Full survey and **(b)** V 2p.

As shown in Figure S8a, the as-prepared KVO film contains V, K, and O elements (excluding C), which further confirms the successful intercalation of K between the V_3O_8 interlayers. Figure S8b depicts the high-resolution V 2p core-level XPS spectra of the KVO film under different color states, indicating that the electrochromic properties of the KVO films are achieved by the chemical valence change of V at different voltages. The most intense doublet peaks, observed at 517.4 and 524.1 eV, are assigned to the V^{5+},[3] whereas the two peaks centered at 516.65 and 522.21 eV correspond to the V^{4+}. As shown in the XPS V 2p spectra of the as-annealed KVO electrode, the ratio of V^{4+}/V^{5+} is 1.7, indicating that this electrode is not fully oxidized. After being charged at 2.0 V, the ratio of V^{4+}/V^{5+} is 0.14, meaning that an electrochemical process is further oxidizing the KVO electrode. When the KVO electrode is changed from a charged state (2.0 V charged) to a discharged state (0.2 V discharged), the V^{4+}/V^{5+} ratio increases from 0.14 to 4, which confirms that Zn^{2+} intercalation results in a reduction of the KVO electrode.

Table S3. The ratio of V^{4+} and V^{5+} in the reduced (0.2 V), oxidized (2.0 V), and as-annealed KVO electrode.

Sample	The atomic percentage of V in different valence states		V^{4+}/V^{5+}
	V^{4+}	V^{5+}	
0.2 V	0.8	0.2	4
2.0 V	0.12	0.88	0.14
As-annealed	0.67	0.37	1.7

Note S1: Calculation process of Zn^{2+} content in an SVO electrode

Firstly, the XPS spectrum is subjected to a peak splitting operation by the software Avantage to obtain the corresponding areas of the Zn 2p peaks. Secondly, the normalized area of the Zn 2p peaks is calculated according to the equation:

Normalized Area [Area (N)] = Peak Area / (SF * TXFN * ECF) **(1)**

TXFN = transmission function

SF = sensitivity factor

ECF = energy compensation factor

According to Equation **(1)**, the normalized area of Zn 2p in the reduced and oxidized SVO electrode is calculated as 75.04 and 68.01, respectively. Therefore, only ~ 9.37% of the inserted Zn^{2+} are extracted by applying a 2.0 V external voltage.

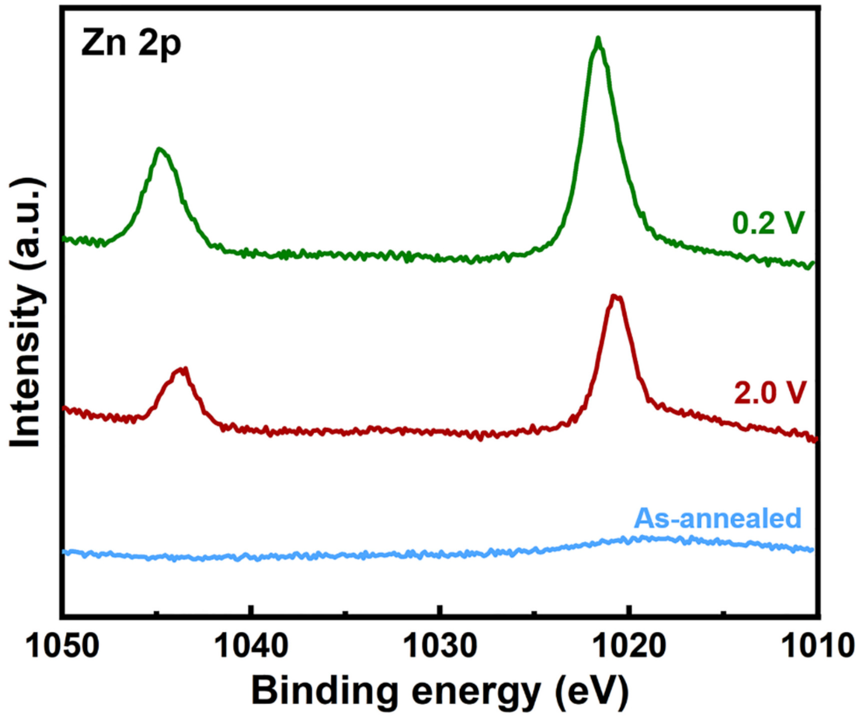

Figure S9. The ex-situ XPS survey spectra of Zn in the SVO electrode after being discharged/charged.

The refined test of the XPS spectrum for Zn in SVO shows that the intercalation behavior of Zn^{2+} accompanies the reduction process of SVO. After the oxidation process of SVO (bias of 2.0 V), the Zn 2p peaks remain in the spectrum, albeit weaker than those following the reduction process (bias of 0.2 V). Such an observation indicates that SVO suffers a great challenge pertaining to "dead Zn^{2+} sites".

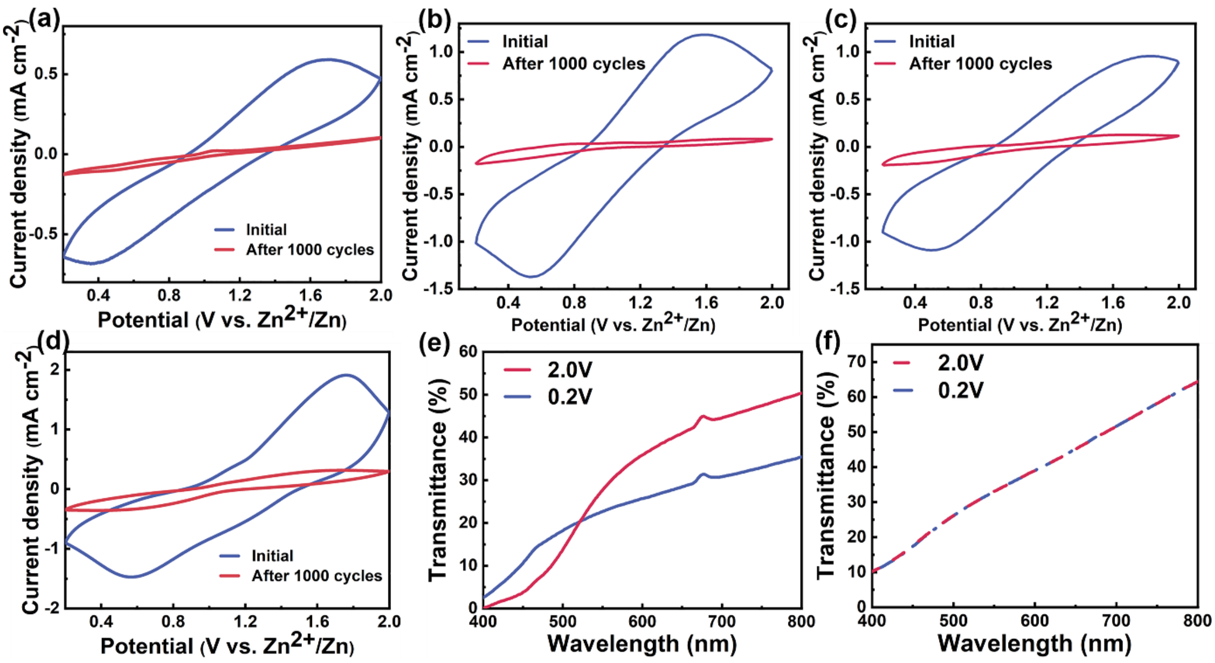

Figure S10. CV curves of the KVO electrode in **(a)** a pure aqueous electrolyte, **(b)** an aqueous electrolyte containing 1wt% PVA, and **(c)** an aqueous electrolyte containing 3wt% PVA. **(d)** CV curves of the SVO electrode in a hybrid electrolyte. **(e)** Optical transmittance of the SVO electrode in the hybrid electrolyte after 1000 cycles. **(f)** Optical transmittance of the KVO electrode in aqueous electrolytes after 1000 cycles.

The electrochemical cyclic voltammetry cycle stability is measured at 150 mV s^{-1} between 0.2 and 2.0 V. As shown in Figure S10a, the KVO electrode in a pure aqueous electrolyte retains no capacity after 1000 cycles. The KVO electrode in the aqueous electrolyte containing 1wt% and 3wt% PVA retains 7.82% (Figure S10b) and 13.6% (Figure S10c) of the initial capacity, respectively, after 1000 cycles. Although the stability of the KVO electrode is improved by increasing the PVA concentration, it is still far behind the stability of KVO electrodes when using an ether-water hybrid electrolyte (Figure 3h). When considering the SVO electrode in the hybrid electrolyte, it only remains 15.38% of the initial capacity after 1000 cycles (Figure S10d). The transmittance spectrum clearly indicates that the KVO electrode does not exhibit electrochromic performance after 1000 cycles in a pure aqueous electrolyte (Figure S10f), while the SVO electrode shows severe electrochromic performance degradation after 1000 cycles (Figure S10e).

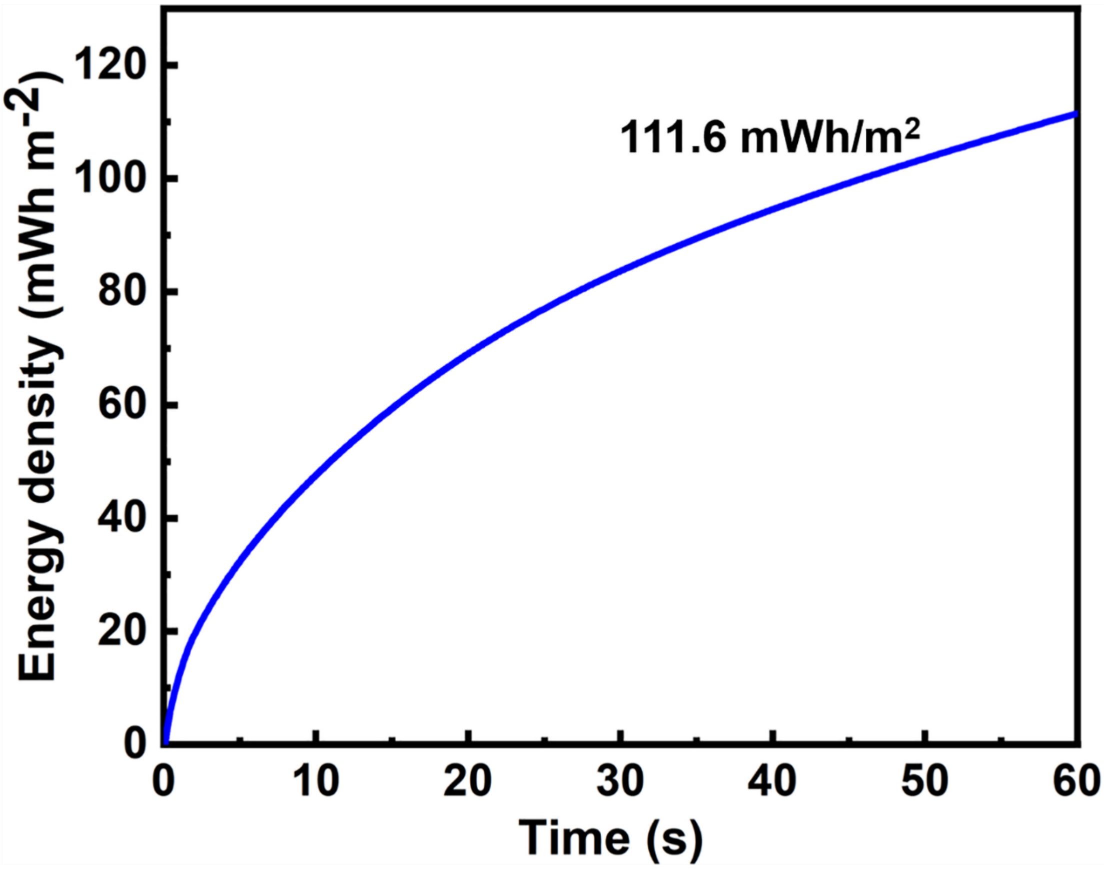

Figure S11. Energy density characteristics of the Zn-KVO display as determined by applying a voltage of 2.0 V for 60 s.

To calculate the input energy density of the Zn-KVO display, a voltage of 2.0 V is applied for 60 s. As shown in Figure S11, the energy density profile indicates that the bleaching process of the Zn-KVO display requires 111.6 mWh/m^2.

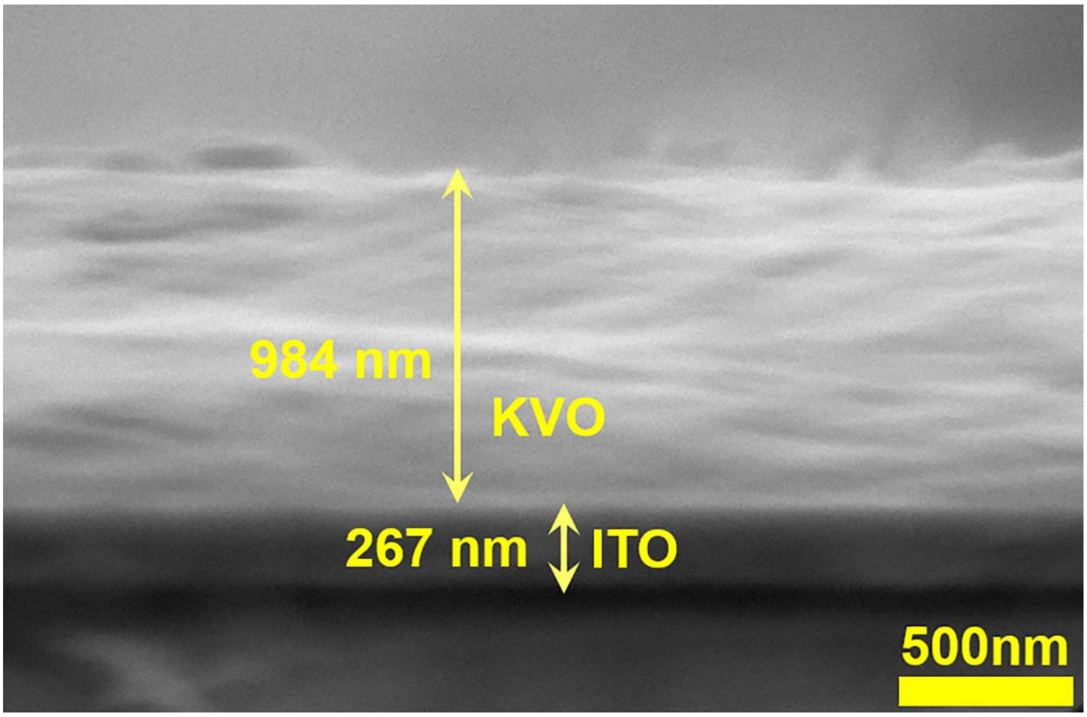

Figure S12. The cross-section SEM image of the KVO electrode.

References

[1] S. Liu, H. Zhu, B. Zhang, G. Li, H. Zhu, Y. Ren, H. Geng, Y. Yang, Q. Liu, C. C. Li, *Adv. Mater.* **2020**, *32*, 2001113.

[2] P. Lei, J. Wang, Y. Gao, C. Hu, S. Zhang, X. Tong, Z. Wang, Y. Gao, G. Cai, *Nanomicro Lett.* **2023**, *15*, 34.

[3] W. Zhang, H. Li, M. Al-Hussein, A. Y. Elezzabi, *Adv. Opt. Mater.* **2019**, *8*, 1901224.
